# Systemic sclerosis-associated pulmonary arterial hypertension and pulmonary fibrosis: exploring biomarker discriminators with advanced omics in a Caucasian cohort

**DOI:** 10.3389/fimmu.2026.1755076

**Published:** 2026-02-02

**Authors:** Nada Mohamed-Ali, Vanessa Acquaah, Maneera Al-Jaber, Rikesh Bhatt, Ibrahim Al-Mohannadi, Konduru Seetharama Sastry, Alka Beotra, Daniel Knight, Christopher Denton, Voon Ong, Maryam Ali Al-Nesf, David Abraham, Mohammed Al-Maadheed, Markella Ponticos, Vidya Mohamed-Ali

**Affiliations:** 1 Centre of Metabolism and Inflammation, Department of Inflammation and Rare Diseases, Division of Medicine, University College London, London, United Kingdom; 2 Centre for Rheumatology, Department of Inflammation and Rare Diseases, Division of Medicine, University College London, London, United Kingdom; 3 Anti-Doping Lab Qatar, Doha, Qatar; 4 Department of Cardiac MRI, Royal Free London NHS Foundation Trust, London, United Kingdom; 5 Institute of Cardiovascular Science, University College London, London, United Kingdom; 6 Allergy and Immunology Division, Department of Medicine, Hamad Medical Corporation, Doha, Qatar

**Keywords:** biomarkers, metabolomics, proteomics, pulmonary fibrosis, pulmonary hypertension, systemic sclerosis

## Abstract

**Introduction:**

Systemic sclerosis (Scleroderma; SSc) is associated with high morbidity and mortality, particularly in patients with pulmonary arterial hypertension (SSc-PAH) and pulmonary fibrosis (SSc-PF). Effective risk stratification and treatment of SSc remains a significant challenge. This proof-of-concept study aimed to identify potential biomarkers capable of distinguishing between three SSc patient groups, defined by no pulmonary involvement (SSc-NLD; n=30), SSc-PAH (n=30), SSc-PF (n=30) compared to healthy controls (HC; n=30).

**Methods:**

The study employed Olink-based proteomics using the Cardiovascular II and Immuno-oncology panels, and untargeted metabolomic profiling using Ultra-high Performance Liquid Chromatography-Tandem Mass Spectroscopy (UPLC-MS/MS), to discover distinct molecular signatures.

**Results:**

Proteomics analysis revealed significantly elevated levels of MCP-1, MCP-3, and MCP-4 in SSc-PF compared to all other groups. However, no robust discriminatory cytokines were identified for SSc-PAH or SSc-NLD. Validation of systemic MCP-1 and IL-6 by ELISA supported the proteomics findings. IL-33 levels were found to be reduced in the SSc-PAH group. Increased levels of pro-inflammatory sIL-6R were also identified in SSc-PAH and SSc-PF, indicating shared inflammatory pathways. Protein-protein interaction analyses demonstrated greater network complexity in SSc-PF, with pathway analysis suggesting overlapping biological mechanisms across pulmonary groups. Metabolomics analysis uncovered a unique panel of metabolites altered exclusively in SSc-PAH, including quinolinate, dimethylarginines, hydroxyasparagine and orotidine. In contrast, no metabolites were uniquely discriminatory for SSc-PF or SSc-NLD. Metabolite-metabolite interaction networks revealed nicotinate and nicotinamide metabolism as the more significantly enriched metabolic pathways in SSc-PAH. Correlation analyses identified distinct protein-metabolite profiles across groups. Of note is the loss of IL-33-related metabolic associations specific to SSc-PAH.

**Discussion:**

This study identified a candidate biomarker panel comprising three cytokines and ten metabolites capable of differentiating between SSc-PAH, SSc-PF, SSc-NLD, and HC. Biomarkers of SSc-PAH were linked to nicotinate and nicotinamide, as well as tryptophan metabolism, whereas those of SSc-PF reflected immune cell infiltration and fibrosis. These findings highlight the potential biomarker panels for diagnosis and targeted therapeutic development.

## 1 Introduction

Systemic sclerosis (Scleroderma; SSc) is a chronic, autoimmune disease characterized by vascular damage and activation of the immune system which triggers the excessive production and deposition of extracellular matrix (ECM) proteins. This process is driven by heightened inflammation, autoimmunity, and small vessel vasculopathy promoting abnormal connective tissue remodeling and scarring. These changes lead to progressive fibrosis affecting the skin and internal organs including the heart, kidneys, and lungs. Currently, the highest mortality rates among SSc patients are those with cardiopulmonary manifestations, accounting for ~50% of SSc-related deaths (1–3). Major pulmonary complications are systemic sclerosis-associated pulmonary artery hypertension (SSc-PAH), and systemic sclerosis-associated pulmonary fibrosis (SSc-PF) (4–6). SSc-PAH and SSc-PF are distinct disease endotypes, involving common and unique pathologic mechanisms that are not yet fully understood, leading to distinct clinical associations, predictive factors, and treatment regimens (7, 8).

SSc-PAH results from the extensive remodeling of the small pulmonary arteries, resulting in a narrowed lumen and increased pulmonary vascular resistance. The pathogenesis has been attributed to an imbalance of vasoactive mediators, pro-inflammatory cytokines, dysregulated thrombosis and disruption of several pathways, including cytokines involved in the Transforming Growth Factor-beta (TGF-β) signaling pathway. This results in altered endothelial and smooth muscle cell proliferation within the pulmonary arterioles (9, 10). There is also an indication that SSc-PAH patients exhibit distinctive, unfavorable metabolic profiles, which are attributable to oxidative stress and mitochondrial dysfunction (11, 12).

SSc-PF is an interstitial lung disease characterized by inflammation, fibrosis, and deposition of ECM within the lung parenchyma leading to diminished gas exchange and decreased vital lung capacity (5). Endothelial cell injury with subsequent vascular damage and alveolar epithelial cell injury are key initial insults that precede fibrosis. Upon injury, pro-inflammatory and pro-fibrotic mediators are released, that activate interstitial fibroblasts (13). Over time fibroblasts acquire features of smooth muscle cells and become myofibroblasts, resulting in dysregulated accumulation of collagen and other ECM components and ultimately leading to fibrosis (14). Cytokines and growth factors, such as TGF-β, interleukin-6 (IL-6) and connective tissue growth factor (CTGF), have all been implicated in the pathogenesis of the disease (15–17). Targeted anti-inflammatory and anti-fibrotic therapies such as tocilizumab, mycophenolate mofetil and nintedanib have been shown to improve patient prognosis, however these lack disease specificity (18, 19).

In the early stages of disease, the pathogenesis of SSc-PAH and SSc-PF appear to involve common mechanisms including vascular dysfunction and inflammatory processes, creating a disparity between mediators and autoimmunity (20–22). These are likely to reflect some shared factors whose overlapping roles are not completely understood, yet lead to distinct features as the diseases progress. SSc-PF can also contribute to pulmonary hypertension in SSc, due to chronic hypoxia and vessel damage from fibrosis in advanced interstitial lung disease (23). This complex overlapping of disease pathogenesis makes risk stratification, early intervention and management challenging. Thus, robust methods to predict the onset and nature of pulmonary complications associated with SSc would help in guiding early diagnosis and intervention. Moreover, specific discriminatory markers between SSc-PAH and SSc-PF could allow for the identification of new disease mechanisms and therapeutics targeting the underlying causes.

In recent years, omics-approaches including transcriptomics, proteomics and metabolomics have made significant advances in understanding disease pathogenesis and in detecting new biomarkers and therapeutic targets (24–26). A transcriptomic study of SSc-PF patients detected abnormalities in levels of IL-6, insulin growth factor binding protein-2 (IGFBP-2), insulin growth factor like-2 (IGFL-2) and toll-like receptor-8 (TLR-8) (27). In SSc-PAH, proteomics has identified changes in collagen IV, endostatin, IGFBP-2, IGFBP-7, matrix metallopeptidase-2 (MMP-2), neuropilin-1, N-terminal pro-brain natriuretic peptide and RAGE (28). More recently, using bioactive metabolomics analysis, metabolites of fatty acid oxidation, eicosanoid metabolism, and sex hormones were reported to distinguish between SSc-PAH and IPAH (29). However, to date, there have been no studies using a multi-omics approach to identify markers of SSc-PAH and SSc-PF within SSc cohorts. Therefore, the aim of this study was to use proteomics and metabolomics, to uncover systemic proteome factors and metabolites with the potential to differentiate between SSc-PAH and SSc-PF patients.

## 2 Materials and methods

### 2.1 Ethical approval and study design

The study was approved by UK nationally registered ethical committee (REC ref. 6398) and the experiments were conducted in accordance with national research guidelines.

### 2.2 Study recruitment

Participants were recruited from the Royal Free Hospital Scleroderma Centre, a national referral center with a database of over 2000 SSc patients and around 1000 control subjects. The SSc cohort consisted of 120 Caucasian participants categorized into four groups of equal numbers (n=30 per group): SSc-PAH, SSc-PF, SSc-NLD and HC.

### 2.3 Inclusion criteria for participant selection

All SSc patients recruited were diagnosed according to the EULAR 2013 classification (30). Stringent clinical criteria were used to ensure the four study groups were clearly defined. All patients selected were European Caucasian to limit population differences. Patients were also selected depending on their autoantibody profiles, as 95% of SSc patients have detectable autoantibodies which, in most cases, are specific for the disease (31) (**Table 1**).

**Table 1:** Cohort demographics and clinical characteristics.

Features	HC (n=30)	SSc-NLD (n=30)	SSc-PAH (n=30)	SSc-PF (n=30)
Age (years)	46.1 ± 19.4	56.9 ± 8.6	59.9 ± 9.3	66.0 ± 11.6
Male: Female	20:10	3:27	2:28	7:23
Disease duration (years)	NA	10 ± 4.7	8 ± 6.6	8 ± 4.7
Autoantibodies ACA n (%)	NA	16 (53.3)	30 (100)	0 (0)
Autoantibodies ATA n (%)	NA	14 (46.7)	0 (0)	30 (100)
Limited Subset n (%)	NA	24 (80)	30 (100)	23 (76.6)
Diffuse Subset n (%)	NA	6 (20)	0 (0)	7 (23.3)

Data are shown as mean ± SD, or n (%). HC, Healthy controls; SSc-NLD, Systemic sclerosis with no lung disease; SSc-PAH, Systemic sclerosis with pulmonary arterial hypertension; SSc-PF, Systemic sclerosis with pulmonary fibrosis; ACA, Anti-centromere autoantibodies; ATA, Anti-topoisomerase autoantibodies; NA, Not applicable. Groups were not significantly different (p=0.36) by age.

SSc-PAH participants were selected based on 2015 ESC/ERS Guidelines for PAH diagnosis and management with a mean pulmonary artery pressure (mPAP) >25 millimeters of mercury (mmHg) (32). A pulmonary artery wedge pressure (PAWP) of ≤15mmHg and a pulmonary vascular resistance (PVR) of >3.0 wood units (UW), measured using a right heart catheter were also used to confirm the presence of SSc-PAH. Only subjects positive for anti-centromere antibodies (ACA) and absence of significant pulmonary fibrosis were recruited.

SSc-PF patients were classified as those with >30% fibrosis of the lung when measured using high resolution computed tomography (HRCT) or 10-30% fibrosis of the lung with a forced vital capacity (FVC) <70% (33, 34). For this group, anti-topoisomerase (ATA) positive patients with an absence of significant pulmonary hypertension (measured on an echocardiogram using the tricuspid regurgitation velocity (TR vel) were selected (with cut-off value of peak TR vel<2.8 m/s) (35).

Patients in the SSc-NLD group had no evidence of pulmonary fibrosis or pulmonary arterial hypertension, as defined above, and had a disease duration of at least five years, reflecting the typical timeframe for the development of pulmonary complications (33). SSc-NLD patients could be either ATA or ACA positive. HC participants had no diagnosis of SSc or any other known disease and were not blood relatives of individuals with SSc. For the SSc-PAH and SSc-PF groups, the earliest available sample following diagnosis of pulmonary complications was analyzed.

### 2.4 Blood sampling and processing

Plasma and serum were extracted from participant’s whole blood samples. Blood (25ml) was taken from consenting participants using a 20-gauge needle, and serum and plasma (EDTA) were separated by centrifugation at 3000 rpm (17000 x g) for 10min at room temperature and stored at -20°C until analysis. Plasma was used for proteomic and metabolomic analyses, while serum samples were used for ELISA validation and investigation of IL-6 trans signaling and CTGF levels.

### 2.5 Olink proteomics analysis

Plasma protein levels were measured using the Olink Proximity Extension platform (Olink proteomics AB, Uppsala Sweden). The Cardiovascular II and Immuno-oncology 96 plex immunoassay panels were used, as they included the best selection of cytokines that are implicated in cardiovascular, inflammatory, metabolic diseases and oncology. Each panel consisted of 92 proteins (n=184). Duplicated panel analytes (n=17) were removed from the Immuno-oncology panel prior to analysis, leaving a total of 167 analytes. Protein levels were reported as Olink normalized protein expression (NPX). NPX values were calculated from cycle threshold (Ct) values and data was pre-processed to minimize variations between and within assays. A high NPX value is correlated with a high protein concentration (36).

### 2.6 Enzyme-linked immunosorbent assay

For targeted cytokine analysis, serum concentrations in 120 samples (n=30 per group) of IL-6, IL-33 MCP-1, CTGF, sIL-6R and sgp130 were determined using commercial ELISA (R&D systems, Oxfordshire, UK). The assay procedure was carried out according to the recommended protocols. Assay dilutions were IL-6/1:5; IL-33/no dilution; MCP-1/1:2; sIL-6R, sgp130 and CTGF/1:100.

### 2.7 Protein-protein interactions

Network analysis to identify protein-protein interactions between cytokines altered in each group was performed using the STRING database (https://string-db.org/). The STRING platform assesses direct physical interactions and indirect functional associations from five sources: primary curated databases, text mining, experimental interactions, co-expression interaction analyses or interactions seen in organisms with great homology (37). The network was assessed by node degree (the average number of interactions a protein has in the network), and clustering coefficients (a measure of how connected the nodes in the network are. A high clustering coefficient is representative of a highly connected network). Biologics, either FDA-approved or undergoing clinical trials, targeting these cytokines were included in the network. Pathway analysis was conducted using the gene ontology (GO) database in the Enrichr tool to identify biological and molecular pathways for cytokines altered per group (https://maayanlab.cloud/enrichr-kg).

### 2.8 Metabolomics analysis

A total of 120 plasma samples (n=30 per group) were analyzed by untargeted metabolomics. Sample preparation, processing and QC generation were completed at the Anti-Doping Lab Qatar (ADLQ, Doha, Qatar) while final QC review and curation were completed by Metabolon (Metabolon Inc., Durham, NC, USA). Metabolomics profiling was performed via Ultra-high Performance Liquid Chromatography-Tandem Mass Spectroscopy (UPLC-MS/MS) using established protocols. Raw data were extracted, peak-identified and quality control processed using Metabolon’s hardware and software (26). Compounds were compared to a library of >3300 purified standard compounds. Unknown compounds were also recorded and may be identified in future by acquisition of a matching purified standard or by classical structural analysis (38).

### 2.9 Statistics and bioinformatics

Using R Programming software, a one-way ANOVA with Tukey’s multiple comparisons test and Pearson’s correlations were used to analyze Olink proteomics (R v.4.1.3, R studio build 485). ELISAs utilized Independent-Samples Kruskal-Wallis tests with Dunn’s multiple comparisons test, and Spearman’s Rho correlations (SPSS 7.0). All significantly different proteins per group, as well as cytokines investigated by ELISA, underwent network and pathway analysis. Network analyses were performed using the STRING database. Pathway analysis was conducted using the Enrichr gene ontology (GO) database. Statistical significance was determined using Fischer exact test. Benjamini-Hochberg corrections were applied and a p-value <0.05 was considered significant throughout.

Prior to analysis, unnamed metabolites were excluded from the dataset. Principal Component Analysis (PCA) was performed. Unpaired t-tests were subsequently conducted to compare each group against the remaining three groups. To account for multiple comparisons, the Benjamini-Hochberg correction was applied, with an adjusted p-value <0.01 considered significant. Metabolites were retained only if they exhibited significant dysregulation in the target group compared to all other groups, without showing significant directional dysregulation (up or down) in any other group. Finally, the retained metabolites were validated by training a Random Forest model to confirm their predictive capability. All metabolomics analyses were conducted using Python (v3.10.19) and the scikit-learn library.

Metabolite network analysis and pathway enrichment was performed using the MetaboAnalyst 6.0 software (39). Protein-metabolite correlations utilized Pearson’s correlation test. A p-value <0.05 was used to determine the pathway analysis and correlation significance. All graphs were created either using R software or GraphPad Prism (9.4.0).

## 3 Results

Key cohort demographics and clinical characteristics are shown in **Table 1**.

### 3.1 SSc blood proteome profiling

**Figure 1:**
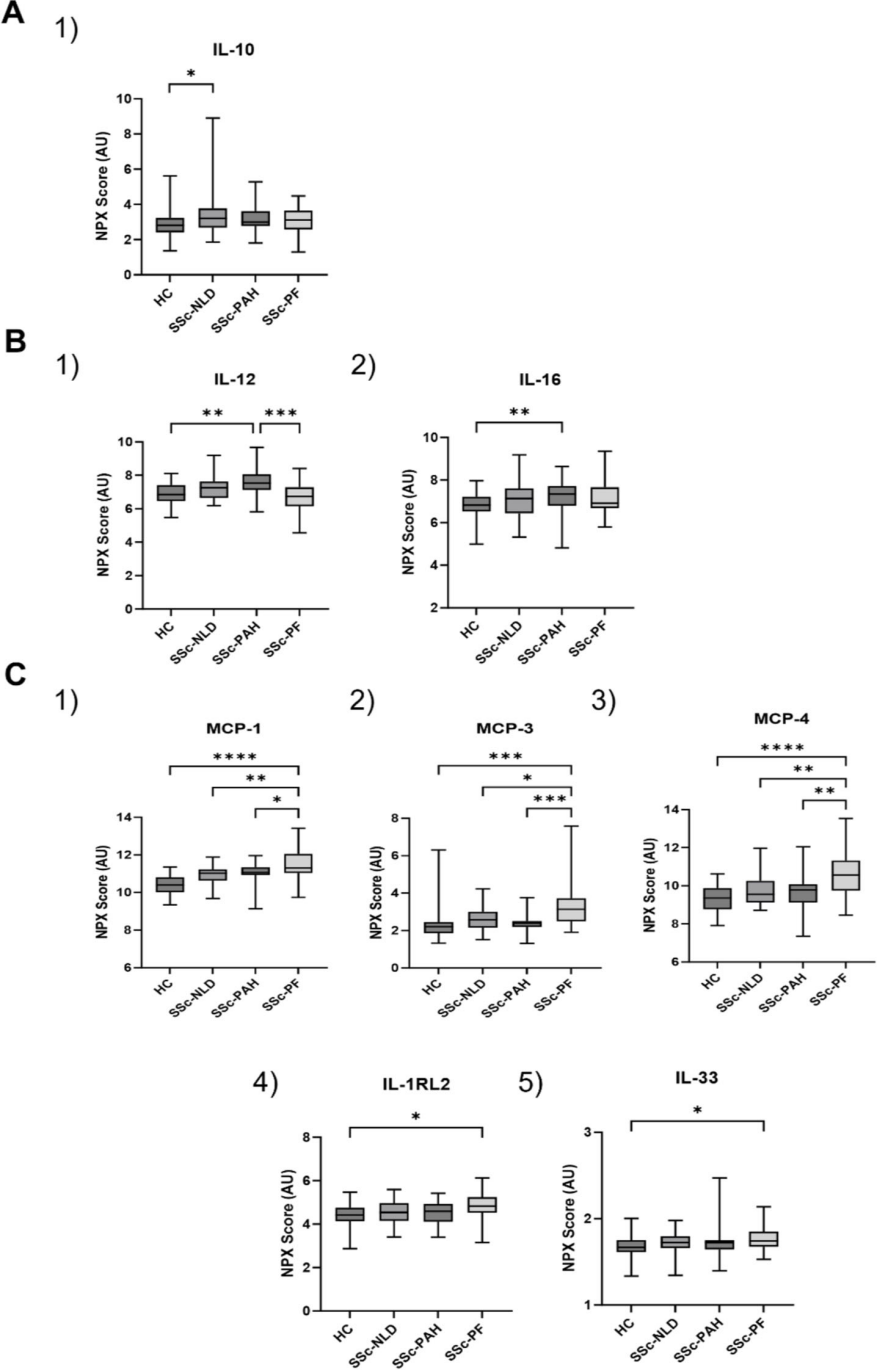
Distinct cytokines are differentially expressed in SSc disease cohorts. Relative cytokine levels identified by proteomic analysis are shown for healthy controls (HC), systemic sclerosis with no lung disease (SSc-NLD), systemic sclerosis with pulmonary arterial hypertension (SSc-PAH), and systemic sclerosis with pulmonary fibrosis (SSc-PF). Data are illustrated as box plots showing the median and interquartile ranges. Cytokines predominantly altered in each subgroup are shown as follows: **(A)** SSc NLD (1. IL-10); **(B)** SSc PAH (1. IL-12, 2. IL-16); and **(C)** SSc PF (1. MCP-1, 2. MCP-3, 3. MCP-4, 4. IL1RL2, 5. IL-33). Statistical significance is indicated by *p<0.05, **p<0.01, ***p<0.001, ****p<0.0001. NPX denotes Olink protein normalization units (AU).

Analysis of plasma samples revealed that the 167 Olink protein analytes were detected in samples examined. Using one-way ANOVA, 12 cytokines were found to be significantly altered in the four groups. These were IL-10, IL-12, IL-16, MCP-1, MCP-3, MCP-4, IL-1RL2, IL-33, IL-6, IL-8, IL-1RA and TNFRSF4. Cytokines found to be significantly elevated in more than one group were excluded from further analysis, as they would be non-specific, leaving 8 cytokines (**Figure 1**). IL-10 was significantly elevated in SSc-NLD compared to HC but not when compared to SSc-PAH or SSc-PF. IL-12 was significantly elevated in SSc-PAH compared to SSc-NLD and HC but not SSc-PF. IL-16 was significantly elevated in SSc-PAH when compared to HC but not SSc-NLD or SSc-PF. MCP-1, MCP-3 and MCP-4 were all significantly and specifically elevated in SSc-PF compared to all other groups, however IL1RL2 and IL-33 were significantly elevated in SSc-PF only when compared to HC.

### 3.2 Validation of selected biomarkers and their inter-relatedness in the SSc sub-sets

**Figure 2:**
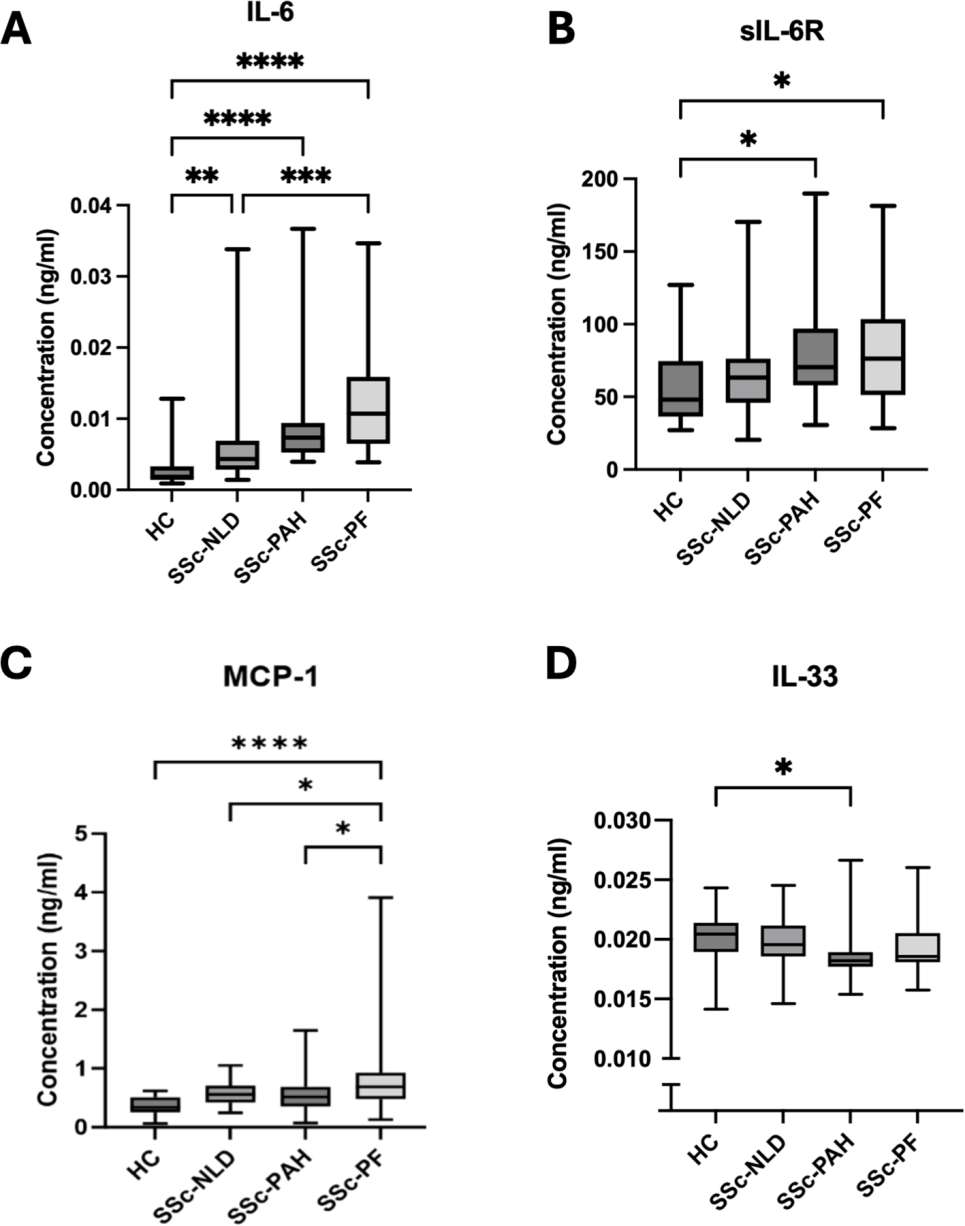
Targeted cytokine profiles in SSc disease cohorts. Cytokine concentrations (ng/ml) identified by ELISA are shown for healthy controls (HC), systemic sclerosis with no lung disease (SSc-NLD), systemic sclerosis with pulmonary arterial hypertension (SSc-PAH), and systemic sclerosis with pulmonary fibrosis (SSc-PF). Data are illustrated as box plots showing the median and interquartile ranges. Cytokines measured were **(A)** IL-6, **(B)** sIL-6R, **(C)** MCP-1 and **(D)** IL-33. Statistical significance is indicated as *p<0.05, **p<0.01, ***p<0.001, ****p<0.0001.

**Figure 3:**
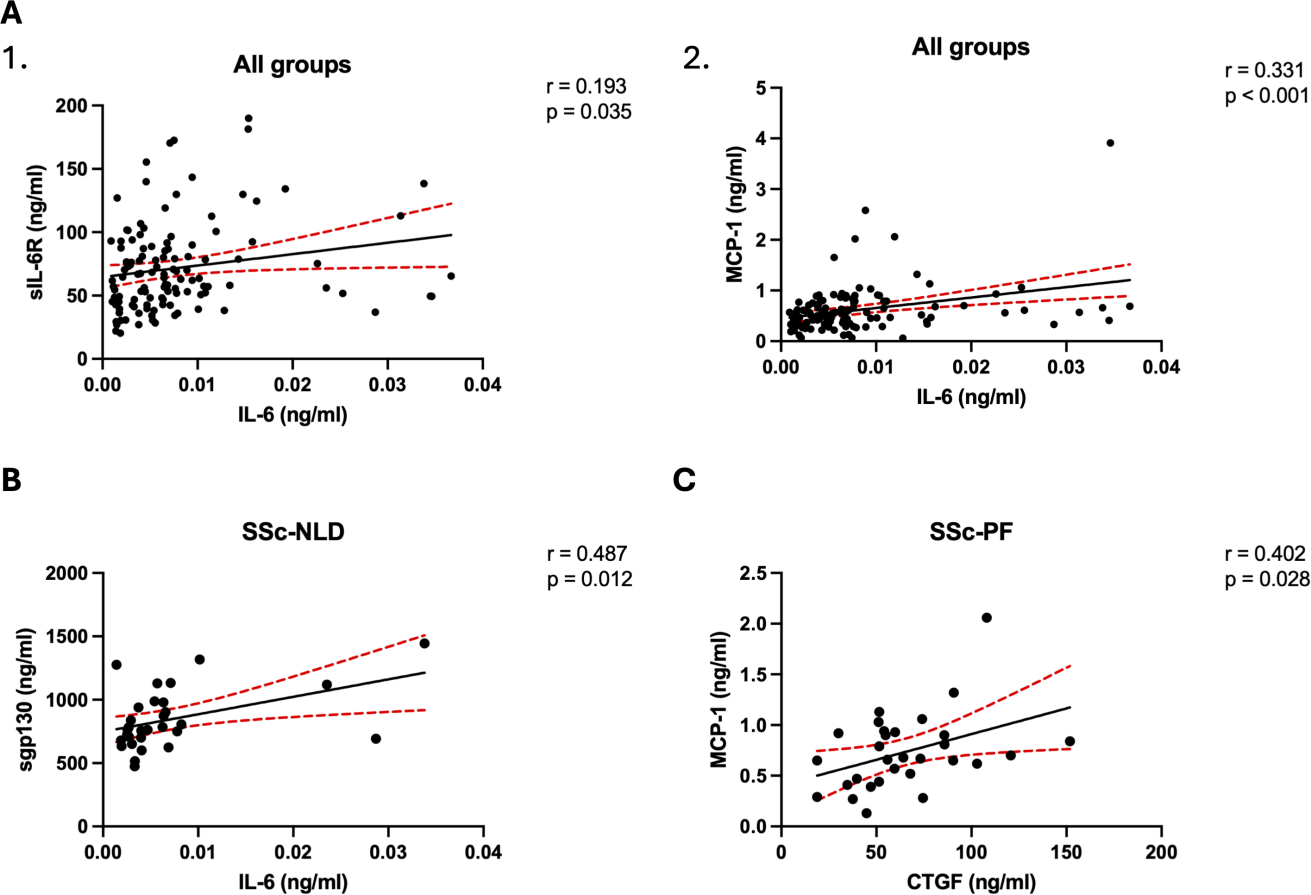
Correlation analysis of targeted cytokines in SSc disease groups. Linear regression analyses of cytokine concentrations (ng/ml) are shown for all groups, systemic sclerosis with no lung disease (SSc-NLD) and systemic sclerosis with pulmonary fibrosis (SSc-PF). Graphs denote significant correlations between IL-6 and sIL-6R **(A1)** and between IL-6 and MCP-1 **(A2)** across the entire cohort. Subgroup analyses demonstrate significant correlations between IL-6 and sgp130 in SSc-NLD **(B)**, and between CTGF and MCP-1 in SSc-PF **(C)**. A p value <0.05 was considered statistically significant. The correlation coefficient (r) and p-value (p) and are shown for each analysis.

Serum levels of IL-6, sIL-6R, sgp130, MCP-1, IL-33 and CTGF were measured by ELISA in the four groups (**Figure 2**). MCP-1 was significantly elevated in SSc-PF compared to all other groups. IL-33 was significantly reduced in SSc-PAH compared to HC. IL-6 was significantly upregulated in all SSc groups compared to HC, and in SSc-PF when compared to SSc-NLD, but no significant difference was found in IL-6 concentrations between SSc-PAH and SSc-PF. With IL-6 trans signaling molecules, sIL-6R was significantly elevated in both SSc-PAH and SSc-PF compared to HC but not when compared to SSc-NLD. There was no significant difference in the levels of the sgp130 and CTGF between groups (data not shown). Interactions between these cytokines were analyzed using Spearman’s correlation analysis (**Figure 3**). When all participants were pooled together, there was a significant positive correlation between IL-6 and sIL-6R, and between IL-6 and MCP-1. In the SSc-NLD group, sgp130 was found to be significantly positively correlated with IL-6. In the SSc-PF group, MCP-1 was significantly positively correlated with CTGF.

### 3.3 Protein-protein interactions and protein pathway analysis in SSc

**Figure 4:**
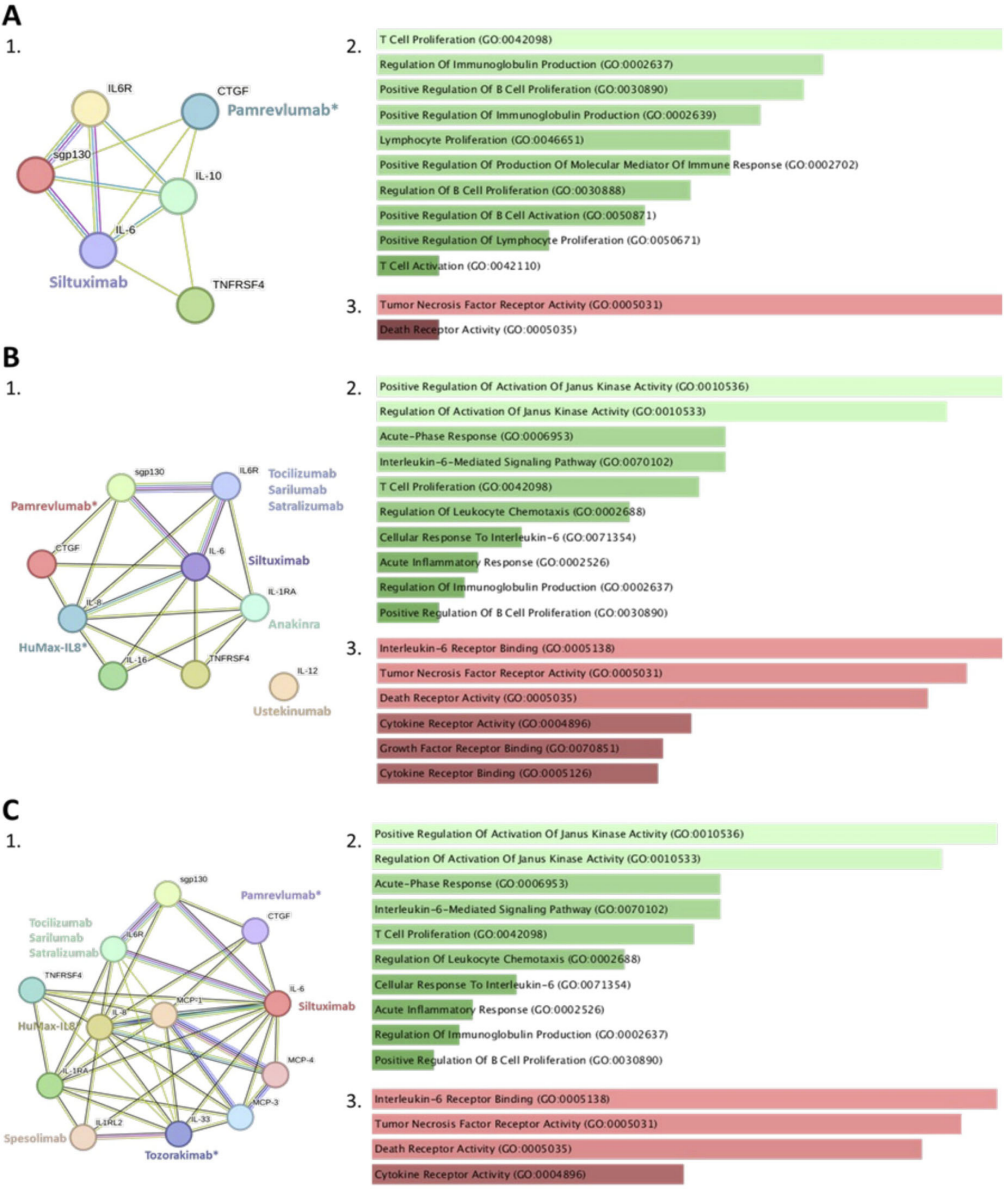
Protein-protein interactions and biological and molecular pathway analysis. Network analysis identified using the STRING database for systemic sclerosis with no lung disease (SSc-NLD; **A1**), systemic sclerosis with pulmonary arterial hypertension (SSc-PAH; **B1**), and systemic sclerosis with pulmonary fibrosis (SSc-PF; **C1**). The network also includes FDA approved biologics and mAbs undergoing clinical trials (*) for cytokines selected for each group. Colored nodes indicate different cytokines, edge colors represent type of evidence: Experimental (purple), Text-mining (yellow), Database (blue) and Co-expression (black). The image illustrates tables of significantly enriched biological pathways for SSc-NLD **(A2)**, SSc-PAH **(B2)** and SSc-PF **(C2)**, with IL-6 related pathways such as T-cell proliferation and Regulation of Janus kinase (JAK) activity being the most significantly enriched. The image also denotes tables of the most significantly enriched molecular pathways for SSc-NLD **(A3)**, SSc-PAH **(B3)** and SSc-PF **(C3)**, with Tumor Necrosis Factor Receptor (TNFR) activity and IL-6 receptor binding being the most significantly enriched. A p value <0.05 was considered statistically significant.

Network analysis identified SSc-NLD as the group with the lowest number of interactions (node degree = 3.67) but a high degree of connectivity between proteins (clustering coefficient = 0.839) (**Figure 4A.1**). The SSc-PAH group had a node degree of 4.0 and had the lowest clustering coefficient (0.713) (**Figure 4B.1**). The SSc-PF group had the highest node degree (6.67) and a clustering coefficient of 0.794 (**Figure 4C.1**). Analysis of current FDA-approved biologics showed that only tocilizumab is routinely used therapeutically in SSc. Several other biologics, such as siltuximab, spesolimab, and anakinra, may potentially be repurposed for treatment of SSc, both in the presence and absence of pulmonary complications (**Figures 4A.1-C.1**).

Pathway analysis identified T cell proliferation and tumor necrosis factor receptor (TNFR) activity as the most significantly enriched biological and molecular pathways in SSc-NLD. For the SSc-PAH and SSc-PF groups, the most significantly enriched pathways were activation of Janus kinase (JAK) activity, and IL-6R binding (**Figures 4A.2.3-C.2.3**).

### 3.4 Untargeted metabolomics

A total of 1189 metabolites were detected, comprising 1001 compounds of known identity (named metabolites) and 188 compounds of unknown structural identity (unnamed metabolites). Unknown metabolites were not included in any further analysis. Additionally, 36 known metabolites were removed due to not fulfilling assay QC criteria. The final analysis performed included 965 known metabolites.

### 3.5 Identification of metabolites and metabolite profiles in SSc

**Figure 5:**
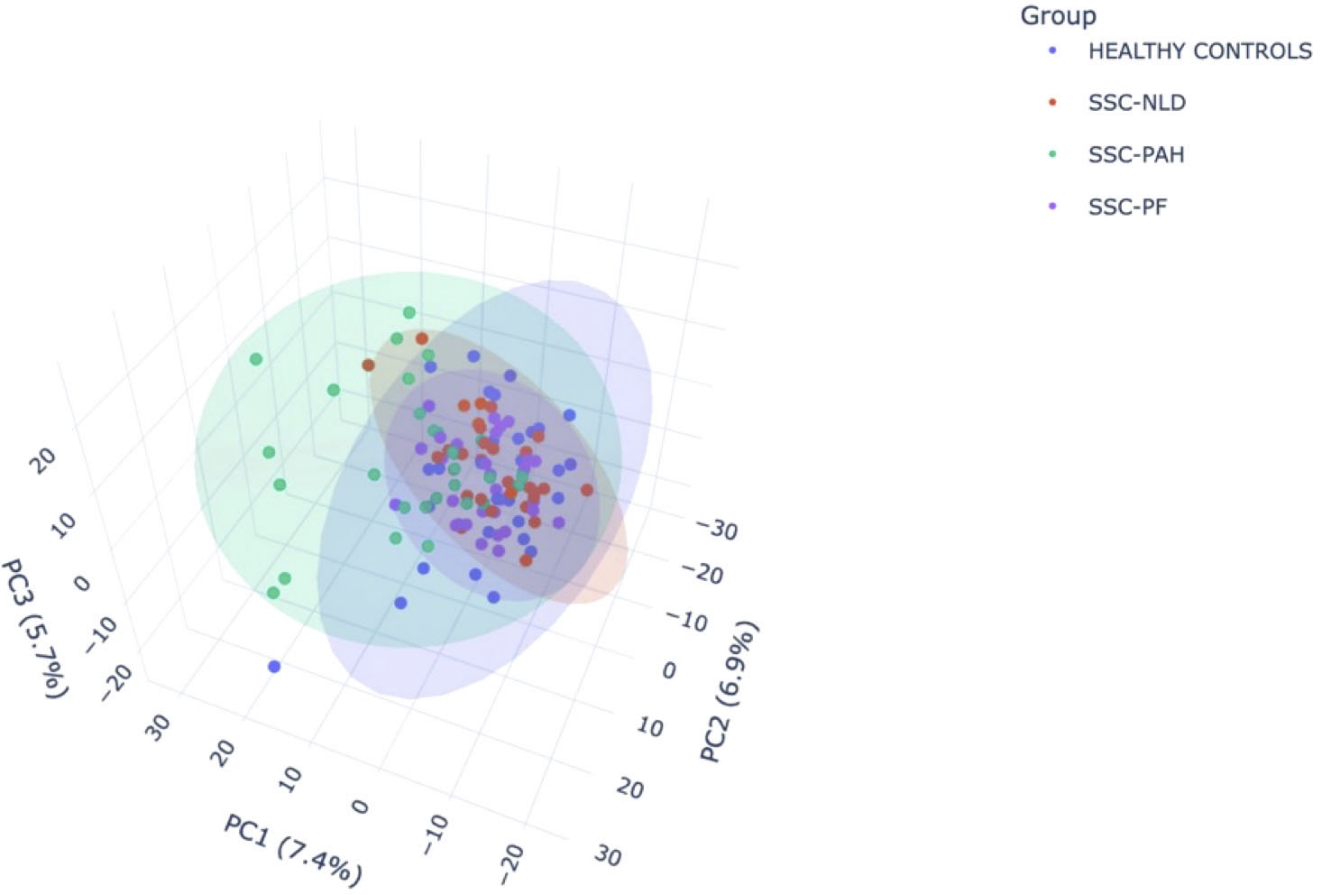
Principal Component Analysis (PCA) 3D score plot of metabolite profiles. The plot illustrates the spatial distribution of samples across the first three principal components (PC1, PC2, PC3), explaining 7.4%, 6.9%, and 5.7% of the variance, respectively. Colored points represent individual samples from the study groups: healthy controls (HC; blue), systemic sclerosis with no lung disease (SSc-NLD; orange), systemic sclerosis with pulmonary arterial hypertension (SSc-PAH; green), and systemic sclerosis with pulmonary fibrosis (SSc-PF; purple). Shaded ellipses indicate 95% confidence regions.

**Figure 6:**
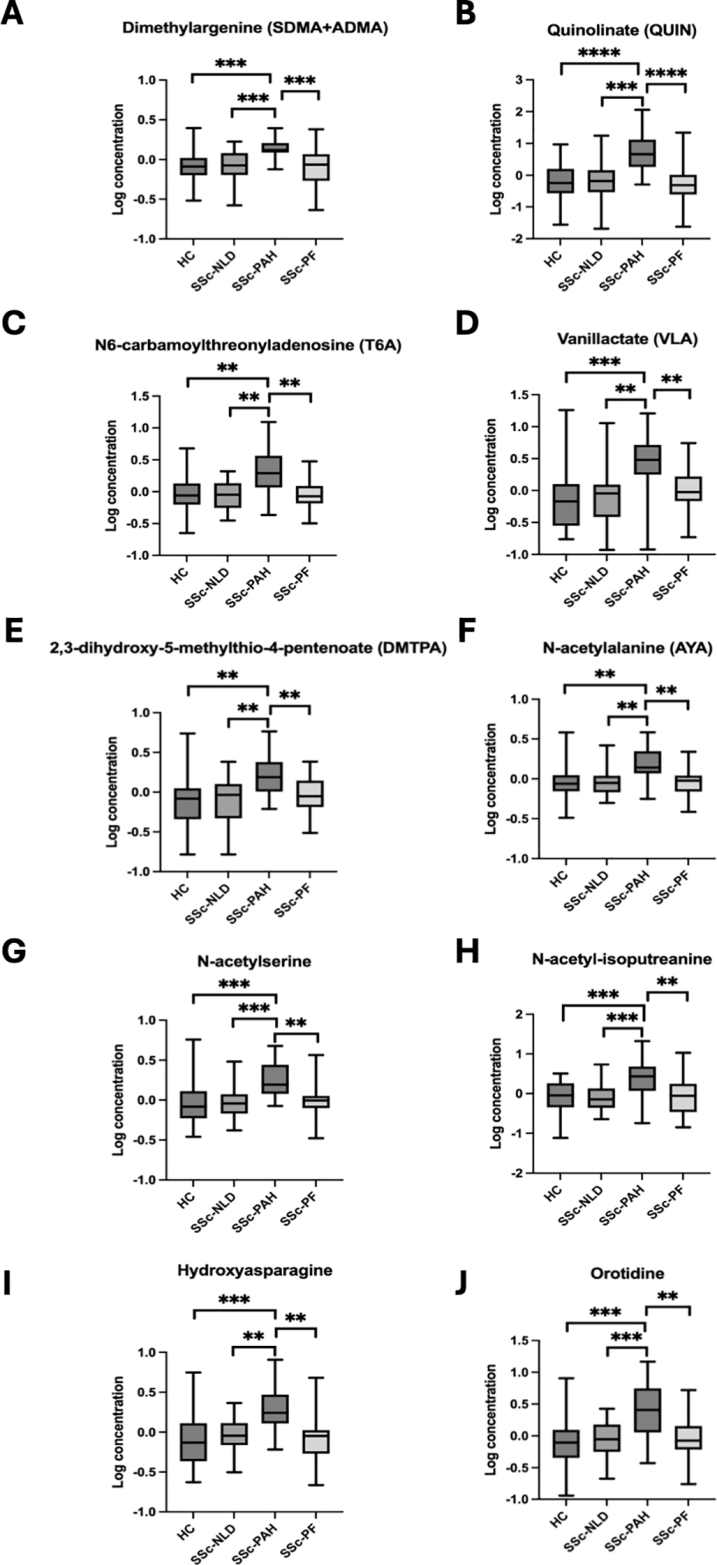
Metabolites selectively increased in SSc-PAH. Relative metabolite levels identified by metabolomics are shown for healthy controls (HC), systemic sclerosis with no lung disease (SSc-NLD), systemic sclerosis with pulmonary arterial hypertension (SSc-PAH), and systemic sclerosis with pulmonary fibrosis (SSc-PF). Data are illustrated as box plots showing the median and interquartile ranges of metabolites significantly elevated in the SSc-PAH group compared with SSc-PF, SSc-NLD and HC. Metabolites include dimethylarginines (SDMA+ADMA; **A**), quinolinate (QUIN; **B**), N6-carbamoylthreonyladenosine (T6A; **C**), vanillactate (VLA; **D**), 2,3-dihydroxy-5-methylthio-4-pentenoate (DMTPA; **E**) N-acetylalanine (AYA; **F**), N-acetylserine **(G)**, N-acetyl-isoputreanine **(H)**, hydroxyasparagine **(I)**, orotidine **(J)**. Statistical significance is indicated as **p<0.01, ***p<0.001, ****p<0.0001.

PCA revealed a modest separation of the SSc-PAH group from the other cohorts, whereas the SSc-PF, SSc-NLD, and HC groups exhibited significant overlap (**Figure 5**). Initial univariate testing identified 122, 37, and 27 differentially expressed metabolites in the SSc-PAH, SSc-PF, and SSc-NLD groups, respectively. However, following the application of strict inclusion criteria to isolate group-specific signatures, only 10 metabolites were retained for the SSc-PAH group, while no metabolites met the criteria for the SSC-PF or SSC-NLD groups (**Figure 6**). The SSc-PAH group exhibited a modest spatial separation from the other cohorts, whereas the SSc-PF, SSc-NLD, and HC groups showed considerable overlap.

**Figure 7:**
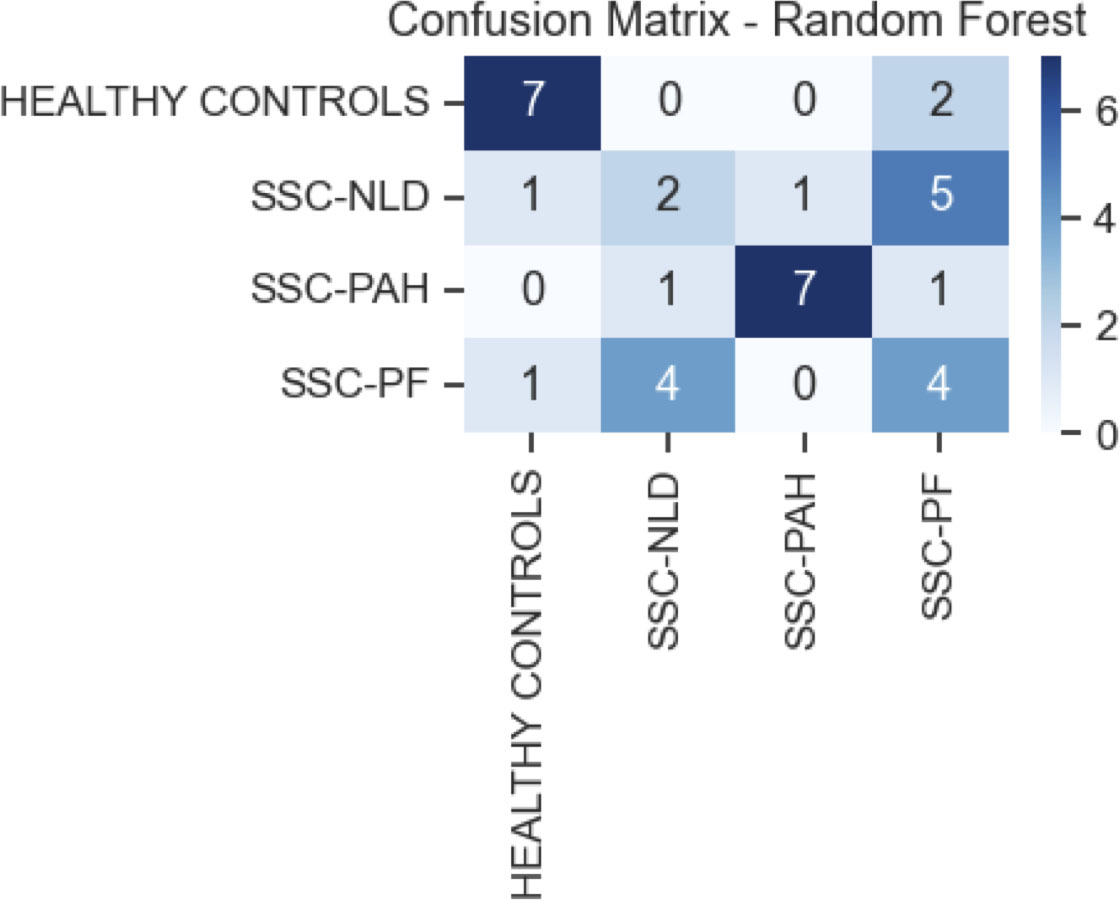
Confusion matrix for the Random Forest classifier. Matrix showing healthy controls, systemic sclerosis with no lung disease (SSc-NLD), systemic sclerosis with pulmonary arterial hypertension (SSc-PAH), and systemic sclerosis with pulmonary fibrosis (SSc-PF). The heatmap displays the classification performance of the Random Forest model trained on the 10 selected metabolites. The y-axis represents the true clinical diagnosis, while the x-axis represents the predicted class. Darker blue cells along the diagonal indicate higher numbers of correct predictions. The cohort was split into training and testing groups - with 21 of each group acting as training group and the remaining (n=9) as the testing cohort.

These 10 retained metabolites were subsequently used to train a Random Forest classifier to evaluate their predictive capability (**Figure 7**). The model demonstrated robust performance in identifying the SSc-PAH group, achieving a precision of 0.70, a recall of 0.78, and an F1-score of 0.74. In contrast, the model exhibited poor predictive power for the remaining groups, with F1-scores ranging from 0.00 to 0.27 (**Table 2**).

**Table 2:** Performance metrics of the Random Forest model for group classification.

Group	Precision	Recall	F1-score	Support
HC	0.33	0.22	0.27	9
SSC-NLD	0.15	0.22	0.18	9
SSC-PAH	0.70	0.78	0.74	9
SSC-PF	0.00	0.00	0.00	9

Precision, recall, and F1-scores reported for the multi-class prediction of systemic sclerosis subgroups and healthy controls. HC, Healthy controls; SSc-NLD, Systemic sclerosis with no lung disease; SSc-PAH, Systemic sclerosis with pulmonary arterial hypertension; SSc-PF, Systemic sclerosis with pulmonary fibrosis. The model was trained using the 10 metabolite features identified as significantly and uniquely dysregulated in the SSc-PAH group. The “Support” column indicates the number of samples per group in the test set. Consistent with the feature selection strategy, the SSc-PAH group shows the highest predictive performance (F1-score = 0.74), while the other groups demonstrate low to negligible predictive accuracy due to the lack of specific markers included in the model.

### 3.6 Metabolite pathway analysis in SSc

**Figure 8:**
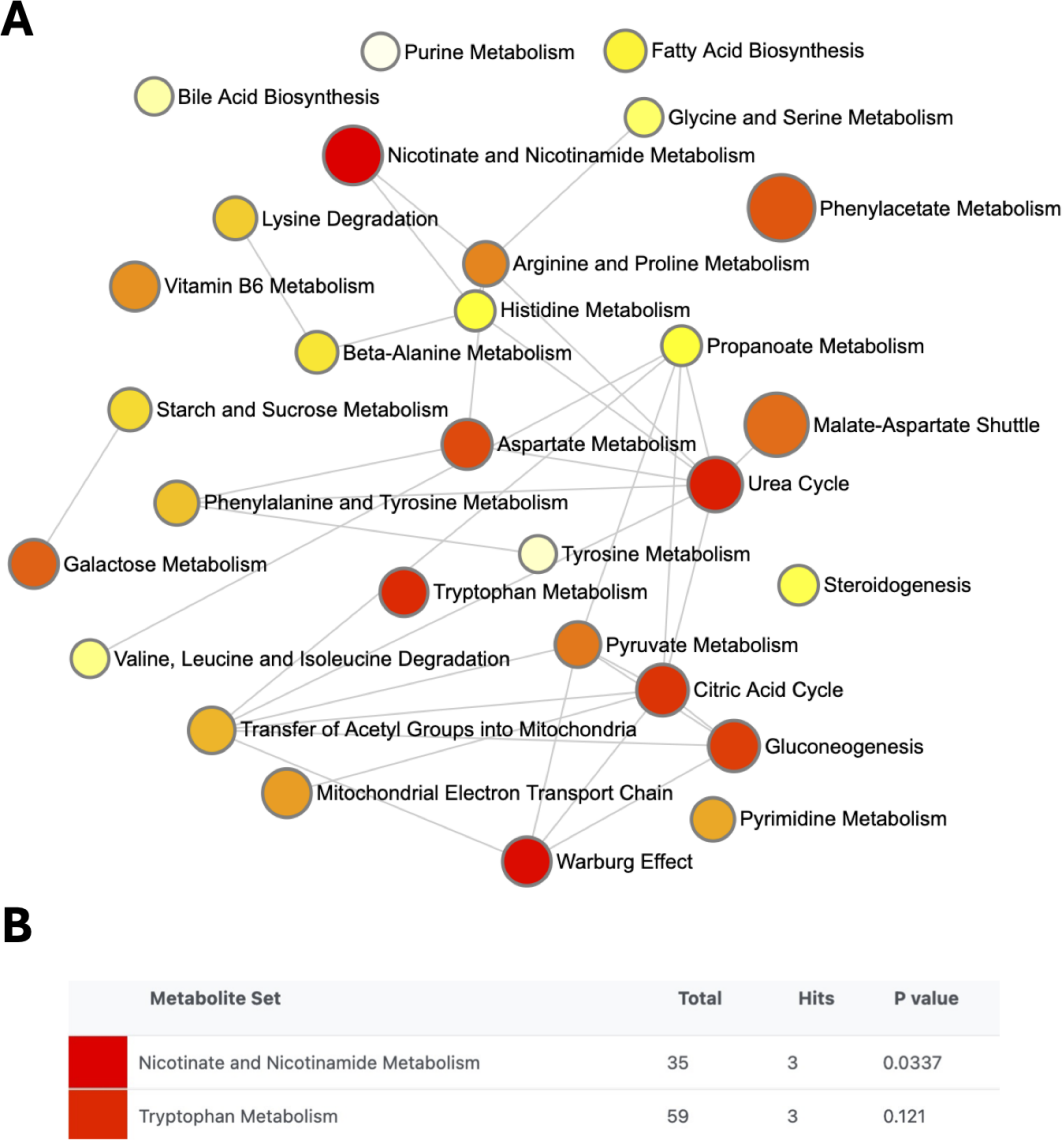
Enriched pathway interaction networks for SSc associated metabolites. Pathway enrichment analysis identified using MetaboAnalyst 6.0 for the systemic sclerosis with pulmonary arterial hypertension (SSc-PAH) group. Enriched pathway network for all metabolites significantly altered in the SSc-PAH group **(A)**. Nodes indicate different metabolic pathways, size of node refers to the extent of enrichment, while the color indicates significance, with lowest p-values (high significance) in red, and highest p-values in yellow (low significance). The nicotinate and nicotinamide metabolism and tryptophan metabolism pathways exhibited the most metabolite hits. A p-value <0.05 was considered significant **(B)**.

Pathway enrichment analysis was performed using all metabolites altered in SSc-PAH to provide greater insight into dysregulated metabolic pathways within this group. An enrichment ratio was calculated by computing hits/expected, where hits = observed hits; expected = expected hits (39). The most enriched pathways in SSc-PAH were those regulating the metabolism of nicotinate and nicotinamide and tryptophan (**Figure 8**). The metabolites involved in these pathways were quinolinate (QUIN), 1-methylnicotinamide, nudifloramide, xanthurenic acid and kynurenine (KYN).

### 3.7 Cytokine-metabolite interactions

**Figure 9:**
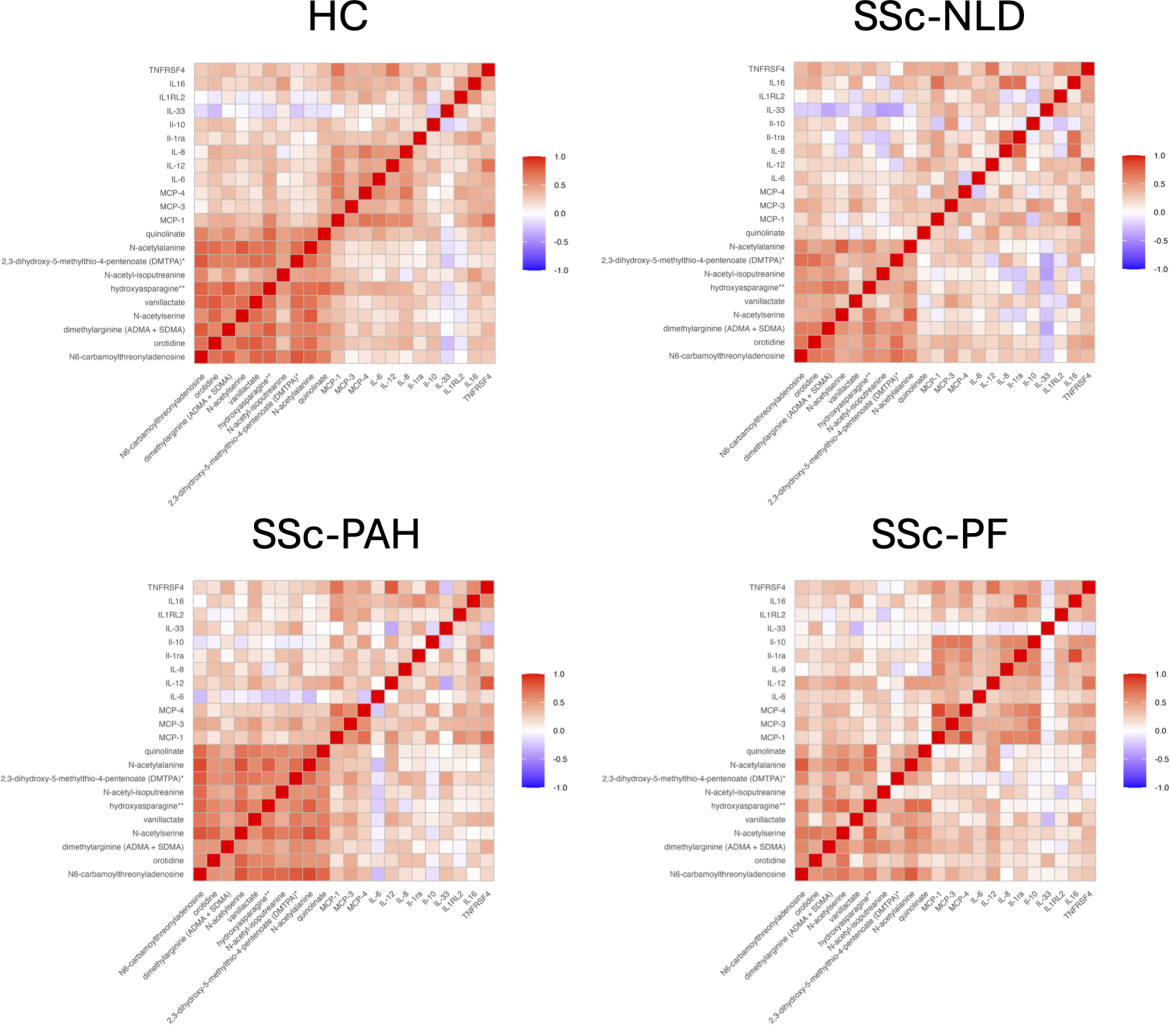
Correlation matrix of cytokines and metabolites. Correlation matrices illustrating relationships between cytokines and metabolites across study groups. Pearson correlation matrices are shown for healthy controls (HC), systemic sclerosis with no lung disease (SSc-NLD), systemic sclerosis with pulmonary arterial hypertension (SSc-PAH), and systemic sclerosis with pulmonary fibrosis (SSc-PF). Red indicates positive correlations and blue indicates negative correlations, with color intensity proportional to correlation strength. A p value <0.05 was considered statistically significant.

Association matrix patterns revealed distinct protein–metabolite profiles that were broadly comparable across the four groups (**Figure 9**). However, a marked disruption in IL-33-related associations was observed in the SSc-PAH group. Of the 10 metabolites analyzed, 9 showed negative correlations with IL-33 in HC and SSc-NLD. These associations were reduced in SSc-PF (4 negatively correlating metabolites) and largely lost in SSc-PAH, with IL-33 exhibiting more positive correlations. Moreover, a negative correlation between IL-33 and IL-12 emerged in the pulmonary groups, which was absent in both SSc-NLD and HC.

## 4 Discussion

Pulmonary complications associated with SSc (SSc-PAH and SSc-PF) are the leading cause of disease-related death. Both conditions have complex etiologies which are not fully understood. Available therapeutics aim to alleviate symptoms, but do not resolve the underlying inflammatory and metabolic dysfunction. Identification of discriminatory biomarkers and novel therapeutic targets would improve patient prognosis and outcomes. This is the first study to produce a combined cytokine-metabolite panel which may potentially distinguish between SSc-PAH and SSc-PF.

MCP-1, MCP-3, and MCP-4 were identified as discriminatory markers for SSc-PF, as their levels were significantly elevated in this group compared to others. These chemokines are members of the monocyte chemoattractant protein family, and are strong chemotactic factors, recruiting immune cells to sites of infection, inflammation, and stress. MCP-1 has been associated with initiation of inflammation in SSc. High levels of its receptor, CCR2, has been detected on CD4^+^ T cells within the fibrotic lung of bleomycin-treated mice (40, 41). Elevated serum MCP-1 has also been shown to correlate with the development of pulmonary fibrosis in SSc patients (42). Similarly, serum MCP-3 levels in SSc patients levels have been associated with greater skin fibrosis and reduced FVC, suggesting a central role in fibrogenesis (43). MCP-4, while not as extensively studied as other members of the MCP family, has been reported to be expressed in cells of the bronchoalveolar lavage and may also contribute to fibrotic disease processes. However, members of the MCP family have also been reported to be elevated in other inflammatory pulmonary conditions, such as idiopathic pulmonary fibrosis (IPF). To date, proteomic studies in IPF have not consistently identified MCPs as established biomarkers. A direct comparison between IPF and SSc associated pulmonary fibrosis would therefore be required to determine whether MCPs represent a robust and disease-specific biomarker (44, 45).

The discriminatory value of MCP-1 between SSc-PAH and SSc-PF was validated in this study by ELISA. IL-33, IL-6, sIL-6R, sgp130 and CTGF, while not found to distinguish between the pulmonary groups, were also investigated. IL-33 levels were found to be reduced in SSc-PAH compared to HC. In trans-signaling, IL-6 bound to its soluble receptor, sIL-6R, and the signal transducer gp130 to promote inflammation, while soluble gp130 blocks this interaction and dampens the response. In this study, IL-6 and sIL-6R were found to be elevated in both SSc-PAH and SSc-PF. However, this may occur through distinct pathogenic pathways in the disease subsets, as suggested by the efficacy of anti-IL-6R therapy with tocilizumab in SSc-PF, but not in SSc-PAH (46, 47). Interestingly, in the SSc-NLD group, there was a significant positive correlation between IL-6 and sgp130. This is potentially a protective mechanism, halting progression and development of pulmonary complications. CTGF has also been implicated in the pathogenesis of SSc. Representational difference analysis (RDA) demonstrated increased CTGF gene transcription in fibroblasts isolated from SSc patients (48). In addition, selective deletion of CTGF in mouse models of PAH and PF significantly reduced pulmonary interstitial scarring and vascular remodeling (49). While no significant differences in CTGF levels were observed across patient groups in this study, there was a strong positive correlation between CTGF and MCP-1 in the SSc-PF group, suggesting that CTGF signaling may contribute to this disease pathway.

Network analysis identified SSc-PF as the group with the highest node degree, indicating that cytokines within this subset exhibited a greater number of interactions with one another. This finding aligns with previous studies suggesting that SSc-PF represents a more inflammation-driven phenotype. Collectively, the cytokines involved and their interconnectedness point to a state of inflammatory dysregulation within this group. Of the seven FDA-approved drugs targeting the cytokines within these networks, four (tocilizumab, sarilumab, siltuximab and satralizumab) target the IL-6 signaling pathway and have been trialed in SSc with varying degrees of success. Anakinra is an anti-IL-1 inhibitor. While increasing evidence suggests that IL-1-mediated inflammation may play a role in SSc-associated cardiac failure, to date, there are no reports of its use in SSc-related disease (50). Ustekinumab targets the p40 subunit, shared by IL-12 and IL-23, and has demonstrated efficacy in immune mediated conditions such as psoriasis. However, there is currently no evidence supporting its use in SSc (51). Spesolimab is a monoclonal antibody targeting the IL-36 receptor and has shown clinical benefit in the treatment of generalized pustular psoriasis; however, its role in SSc has not been investigated (52). Repurposing these drugs may potentially offer a wider range of therapeutic options for patients.

A 10-metabolite signature was uncovered for SSc-PAH. Dimethylarginines (SDMA + ADMA together), Hydroxyasparagine, QUIN, AYA, T6A, VLA, DMTPA, N-acetylserine, N-isoputreanine and Orotidine were significantly elevated in this group compared to all others. These metabolites are important for energy metabolism, mitochondrial oxidation, amino acid catabolism, and inflammation. Dimethylarginine (ADMA) is known to regulate vascular tone, response to injury, and is a known inhibitor of nitrous oxide (NO) synthase. Decreased levels of NO is a key driver of chronic inflammation and cellular disruption, as it leads to endothelial dysfunction. ADMA levels have been shown to be elevated in the plasma of IPAH and chronic thromboembolic pulmonary hypertension (CETPH) patients compared to healthy controls, and this correlated with low endothelial NOS expression (53, 54). Clinically, serum ADMA has been shown to positively correlate with mPAP and pulmonary vascular resistance, and so could serve as a biomarker for monitoring the effectiveness of treatment (55). However, ADMA was not measured separately in this study.

Hydroxyasparagine is important in post-translational modification of calcium-binding epidermal growth factor (cbEGF)-like domains. These domains are present in a wide variety of extracellular proteins such as fibrillin. Fibrillin has been shown to induce the trans differentiation of resident fibroblasts into myofibroblasts, which produce ECM proteins and pro-inflammatory cytokines that exacerbate inflammation (56). This is a key inflammatory pathway involved in regulating chemotaxis of inflammatory cells, which can lead to inflammatory cascades within the vasculature (57).

High levels of QUIN, a metabolite formed through the kynurenine pathway (KP) of tryptophan metabolism, has been associated with lower survival rates in treatment naïve PAH patients. Interestingly, in human lung primary cells, treatment with IL-6/sIL-6R complex lead to similar KP metabolic profiles as in PAH, suggesting that the metabolic dysfunction within the KP is driven by IL-6 trans signaling (58).

N-acetyl-isoputreanine is a by-product in the catabolism and metabolism of spermidine. Spermidine levels have been shown to be elevated in IPAH patients, and may promote proliferation and migration of PASMCs, encouraging vascular remodeling in rat pulmonary hypertensive models (59). Vanillactate (VLA), N-acetylalanine, T6A, DMTPA, N-acetyl-serine and orotidine were also shown to be elevated in the plasma of SSc-PAH patients compared to the other three groups. However, none of these have been well-characterized in PAH, and so further research into the mechanisms of these metabolites is necessary. However, these metabolites may still act as biomarkers if the results are reproduced. The robustness of the identified metabolites were evaluated using Random Forest. The analysis demonstrated that the identification of metabolite markers in the SSc-PAH group was significantly robust compared to other groups.

Nicotinate and nicotinamide metabolism was the most significantly affected metabolic pathway in SSc-PAH. Nicotinate and nicotinamide are precursors of the co-enzyme’s nicotinamide-adenine dinucleotide (NAD+) and NADP +. CD38, which hydrolyses NAD and nicotinamide, is overexpressed in skin of patients with diffuse SSc compared to healthy individuals. Depletion of NAD+ by CD38 impairs the ability for PARP to repair DNA lesions which encourage pro-inflammatory and pro-fibrotic phenotypes (60). Enrichment of the nicotinate and nicotinamide pathways may therefore imply an imbalance of the resolution of inflammation and fibrosis.

In this study, most of the protein-metabolite, protein-protein and metabolite-metabolite correlations were found to be comparable across groups. However, several notable differences were observed. In HC and SSc-NLD groups, IL-33 showed strong negative correlations with most metabolites, consistent with its role in maintaining metabolic homeostasis through ST2-mediated regulation of key metabolic checkpoints (61). These associations weakened in SSc-PF, indicating diminished IL-33–mediated control of immune–metabolic balance, and were completely lost in SSc-PAH, suggesting disruption of this pathway. Reduced systemic IL-33 levels have been reported in idiopathic PAH, suggesting a loss of its protective function. IL-33 has been implicated in cardiopulmonary protection by reducing inflammation and limiting vascular remodeling, mediated primarily via the ST2L receptor. Notably, IL-33 exhibits context dependent activity and can either activate, promoting inflammation, or reduce resolving the inflammatory response. In this context, our findings of reduced IL-33 levels in PAH may reflect a loss of protective IL-33 signaling (62–64). Moreover, IL-33 and IL-12 were not correlated in HC, but positively associated in SSc-NLD, likely reflecting early immune activation where IL-33 release enhances IL-12 production (65). In contrast, the pulmonary groups exhibited a negative correlation, suggesting immune polarization, where chronic IL-33 signaling suppresses IL-12 activity, driving a Th2-dominant, fibrotic environment (66).

In conclusion, this proof-of-concept study proposes a combined cytokine–metabolite signature which may potentially distinguish between SSc-PAH and SSc-PF. While the biomarker panel identified in this study largely aligns with previous findings, it may also hold novel diagnostic, prognostic, and therapeutic potential. However, its clinical applicability requires validation in a larger, independent cohort.

Key limitations of this proof-of-concept study include the small sample size and the absence of detailed information on comorbidities and treatment regimens. Although the cohorts were ethnicity and age matched, there were sex differences between the groups, reflecting the higher prevalence of SSc in women. As a result, potential sex related differences may not have been fully captured in the analysis. Finally, differences in medication in the groups may play a role in the metabolite signatures of each of these groups and the possibility that these metabolite profiles may be, at least in part, a medication-associated phenomenon cannot be discounted.

## Data Availability

The raw data supporting the conclusions of this article will be made available by the authors, without undue reservation.
